# Circulating nucleosomes as a potential cancer biomarker in dogs with splenic nodular lesions

**DOI:** 10.1080/01652176.2024.2399648

**Published:** 2024-09-03

**Authors:** Sara Meazzi, Valeria Martini, Laura Marconato, Marina Aralla, Luca Licenziato, Matteo Olimpo, Paola Roccabianca, Silvia Sabattini, Alessandra Ubiali, Riccardo Zaccone, Luca Aresu

**Affiliations:** a Department of Veterinary Medicine and Animal Sciences, University of Milan, Lodi, Italy; b I-VET, Flero, Italy; c Department of Veterinary Medical Sciences, University of Bologna, Bologna, Italy; d Pronto Soccorso Veterinario Laudense, Lodi, Italy; e Department of Veterinary Sciences, University of Turin, Grugliasco, Turin, Italy

**Keywords:** biomarker, dog, hemangiosarcoma, spleen, histopathology, nodular lesion, plasma, nucleosome

## Abstract

Splenic nodular lesions in dogs can be either benign or malignant. They might be discovered incidentally or, in case of rupture, they may lead to hemoabdomen. Nevertheless, splenectomy followed by histopathology is essential for diagnosis and to prevent rupture. Yet, this invasive procedure might be postponed for dogs with benign splenic nodular lesions. Conversely, owners may opt for euthanasia over surgery for malignancies with poor prognosis like hemangiosarcoma. Thus, anticipating diagnosis with non-invasive biomarkers is crucial for proper patient management. In this prospective study, plasma samples were collected from 66 dogs with histologically confirmed splenic nodular lesions. A canine-specific ELISA kit was applied to assess nucleosome concentration, with histopathology of the spleen serving as the gold standard. Nucleosome concentration was found to be significantly higher in dogs with malignant splenic nodular lesions, particularly in those with hemangiosarcoma and other malignancies. The presence of hemoabdomen, more prevalent in dogs with splenic malignancy, also resulted in increased plasmatic nucleosome concentrations. Plasma nucleosomes could serve as a biomarker for detecting malignant splenic nodular lesions in dogs. More research is needed to understand how nucleosome concentration relate to disease stage and prognosis in dogs with hemangiosarcoma.

Focal or multifocal splenic nodular lesions are frequently observed in elderly dogs (Cleveland and Casale 2016), the majority of which are benign, including hematoma, nodular hyperplasia, and, rarely, myelolipoma (Eberle et al. 2012; Cleveland and Casale 2016; Fernandez et al. 2019). Nodular hyperplasia is characterized by a benign proliferation of resident splenic cells, and can be further categorized into lymphoid, hematopoietic, and complex types, with lymphoid and complex nodular hyperplasia being most frequently diagnosed (Moore et al. 2012; Cleveland and Casale 2016; Sabattini et al. 2018). The classification relies on the predominant cellular component identified histologically, after splenectomy has been performed.

Conversely, malignant splenic nodular lesions arise from different cell types, including smooth muscle, fibrous, nervous, vascular, histiocytic, and lymphoid tissues. In dogs, most common malignant splenic nodular lesions include hemangiosarcoma and, more rarely, lymphoma (Eberle et al. 2012; Cleveland and Casale 2016; Fernandez et al. 2019). Hemangiosarcoma is frequently associated with organ rupture and hemoabdomen carrying a poor prognosis, with a median survival time of 2-3 months for dogs undergoing surgery alone, and 4–6 months if adjuvant chemotherapy is administered (Wood et al. 1998; Hammond and Pesillo-Crosby 2008; Wendelburg et al. 2015; Fernandez et al. 2019; Faroni et al. 2023). Less frequent malignant tumors affecting the spleen include histiocytic sarcoma and stromal sarcoma (Sabattini et al. 2022; Ferrari et al. 2024).

Due to the vascular nature of the spleen, even benign splenic nodular lesions may pose a life-threatening risk, due to their potential rupture and subsequent acute hemoabdomen (Aronsohn et al. 2009). In particular, the differential diagnosis between hematoma and hemangiosarcoma is challenging, since both lesions often go unnoticed until rupture, necessitating emergency surgery, and present with pooled blood in the splenic parenchyma due to hemorrhage or disrupted blood flow, thus adding complexity to clinical and histologic diagnosis (Herman et al. 2019, Schick and Grimes 2022).

The intricate nature of splenic nodular lesions, coupled with the challenge of distinguishing these lesions using ultrasound or even computed tomography (Kutara et al. 2017; Cudney et al. 2021; Millar and Zersen 2021; Burti et al. 2022), underscores the need for advanced diagnostic strategies and comprehensive approaches to improve malignancy detection. Indeed, owners may have to face the decision of whether to pursue surgery without having any prognostic information available. Thus, for a appropriate patient management and owner communication, it would be very useful to anticipate the nature (benign or malignant) of a splenic nodular lesion identified *via* ultrasound even in the absence of signs.

Liquid biopsy methods offer a minimally invasive means of screening for cancer through a blood test, identifying circulating tumor cells, extracellular nucleic acids, exosomes, nucleosomes, and antigens, each with varying sensitivity and specificity (Bao et al. 2024; Flory and Wilson-Robles 2024). Nucleo­somes, composed of a DNA segment wrapped around four core histones, play a vital role in chromatin assembly, DNA protection, and gene regulation across eukaryotic species. Recent studies have shown promising results in defining the plasma nucleosome compartment in several canine cancers, including hemangiosarcoma and lymphoma (Wilson-Robles et al. 2020; Dolan et al. 2021; Wilson-Robles et al. 2021; Wilson-Robles et al. 2022, 2023).

Accurate differentiation between malignant and benign splenic nodular lesions is crucial for timely clinical intervention and treatment planning. Given their frequency and nature, splenic nodular lesions represent a relevant field to explore for the development of nucleosome detection by liquid biopsy, offering a screening method for a more accurate selection among diagnostic and therapeutic options. The various types of splenic nodular lesions, as mentioned, have important clinical implications, as one might opt for vigilant monitoring in the case of benign lesions or splenectomy if malignancy is suspected.

In the current study, we aimed to investigate whether plasma nucleosome levels varied with different splenic nodular lesions in dogs by employing an enzyme-linked immunosorbent assay (ELISA) kit specifically designed for the canine species (Wilson-Robles et al. 2020). This prospective study was designed to enroll dogs with single or multiple benign and malignant splenic nodular lesions for a comprehensive assessment of nucleosome concentrations in diverse clinical conditions, including those with and without hemoabdomen. The hypothesis was that plasma nucleosome concentrations would be higher in dogs with malignancy compared to those with benign splenic nodular lesions.

## Materials and methods

The present study was approved by the Organismo Preposto al Benessere Animale (OPBA), University of Milan, with protocol number 130_2021. Dogs undergoing splenectomy due to focal or multifocal lesions were enrolled from four different Institutions in Italy. Dogs concurrently diagnosed with any systemic, chronic and severe inflammatory disease were excluded from the study. The diagnostic workup to assess concomitant diseases varied among cases, based on the clinical presentation and clinician’s preferences. On the day of surgery, before any drug administration, whole blood was collected from each dog and immediately placed in EDTA tubes. After surgical removal, spleen was collected and processed for histopathology.

Within 40 min of sampling, each EDTA blood tube was centrifuged at 3000 g for 10 min at room temperature, and the resulting plasma was separated keeping the buffy coat layer intact and stored at −20° until processing (for a maximum of 2 months). Plasma samples were thawed to be processed in batches with the Nu.Q^TM^ H3.1 assay (Volition Veterinary, Henderson, NV, USA). The manufacturer’s instructions and protocols previously published were applied (Wilson-Robles et al. 2020), with the exception of storage temperature at −20° instead of −80° until processing.

A pilot study to investigate the impact of sample storage temperature on nucleosome stability was conducted. Briefly, 10 canine blood samples received at the laboratory for various purposes were processed as detailed previously. Each sample was divided into two aliquots: one stored at −20 °C and the other at −80 °C. Both aliquots were kept for the same duration, up to a maximum of 2 months, before processing. Nucleosome concentration was assessed in both aliquots within a single experiment, and the results were compared using Spearman and Passing-Bablok tests. Significant correlation was observed between the results obtained from the −20 °C aliquots and those from the −80 °C aliquots (*p* = 0.001, *r* = 0.867), despite a negative proportional error (slope: 0.2998; 95% CI: 0.0308-0.7090).

For each dog, the following variables were recorded: breed (pure, mixed), sex (female, spayed female, male, neutered male), age (years), weight (kgs), histopathologic diagnosis, hemoabdomen (present, absent), number of lesions (single, multiple), lesion size (only for dogs with single lesion), ongoing therapies (if any).

For statistical purposes, dogs were grouped based on the histopathologic diagnosis into those with malignant and benign splenic nodular lesions. Dogs with splenic malignancy were further subdivided into dogs with hemangiosarcoma and dogs with other malignancies. The nucleosome concentration data were assessed for normality using the Shapiro-Wilk test. Thereafter, Mann-Whitney test was applied to assess potential differences in median nucleosome concentration between groups. Contingency tables were prepared, and the Fisher exact test was applied to assess possible differences in the prevalence of hemoabdomen and larger lesion size between dogs with and without malignancy. All tests were first performed on the entire population of dogs. Subsequently, analyses were stratified based on the presence of hemoabdomen and lesion size (< or ≥ median value of the study population, 60 mm). Differences between dogs with and without hemoabdomen were also assessed using the same test. Finally, a Receiving Operator Characteristics (ROC) curve was drawn and coordinates were used to select the nucleosome concentration most suitable to discriminate between dogs with benign and malignant splenic nodular lesions. Statistical analyses were performed with the dedicated software SPSS v29.0 for Windows (IBM, Somers, NY), and significance was set at *p* ≤ 0.05.

## Results

A total of 66 dogs were enrolled, consisting of 27 (40.9%) mixed breed dogs and 39 (59.1%) purebred, with Labrador Retriever (*n* = 6), German Shepherd (*n* = 5), Golden retriever (*n* = 3), Cocker Spaniel (*n* = 3) and Jack Russell (*n* = 3) being the most frequent breeds. Thirty-five (53.0%) dogs were male (14 neutered) and 31 (47.0%) females (21 spayed). Median age was 11 years (range, 6-16 years) and median weight was 25.6 kgs (range, 5.2-57.0 kgs). Clinical data are listed in Supplementary Table 1.

Eleven (16.7%) dogs were undergoing various therapies at the time of sampling, primarily for concurrent chronic conditions, including dermatopathies (*n* = 2), congestive heart failure (*n* = 2), hypothyroidism (*n* = 2), Addison disease (*n* = 1), chronic kidney disease (*n* = 1), epileptic seizures (*n* = 1), chronic lymphocytic leukemia (*n* = 1), urinary tract infection (*n* = 1), and gastritis (*n* = 1).

At the time of splenectomy, hemoabdomen was present in 34 (51.5%) dogs.

Based on histopathology, 38 (57.6%) dogs had benign lesions, while 28 (42.4%) dogs had cancer. Among the latter, there were 20 hemangiosarcoma, 4 indolent B-cell lymphoma, 2 stromal sarcoma, 1 metastatic adenocarcinoma and 1 anaplastic round cell tumor staining negative for CD3, CD20, IBA-1, MUM-1, CD11d by immunohistochemistry (IHC). Twenty-two (78.6%) dogs with cancer had hemoabdomen, and 6 (21.4%) did not.

Among dogs with benign lesions, 21 (55.3%) had lymphoid nodular hyperplasia, 6 (15.8%) had hematoma, 4 (10.5%) had hematopoietic nodular hyperplasia, 3 (7.9%) had complex nodular hyperplasia, 3 (7.9%) had myelolipoma, and 1 (2.6%) had hemangioma. Twelve (31.6%) dogs had hemoabdomen.

**Figure 1.**
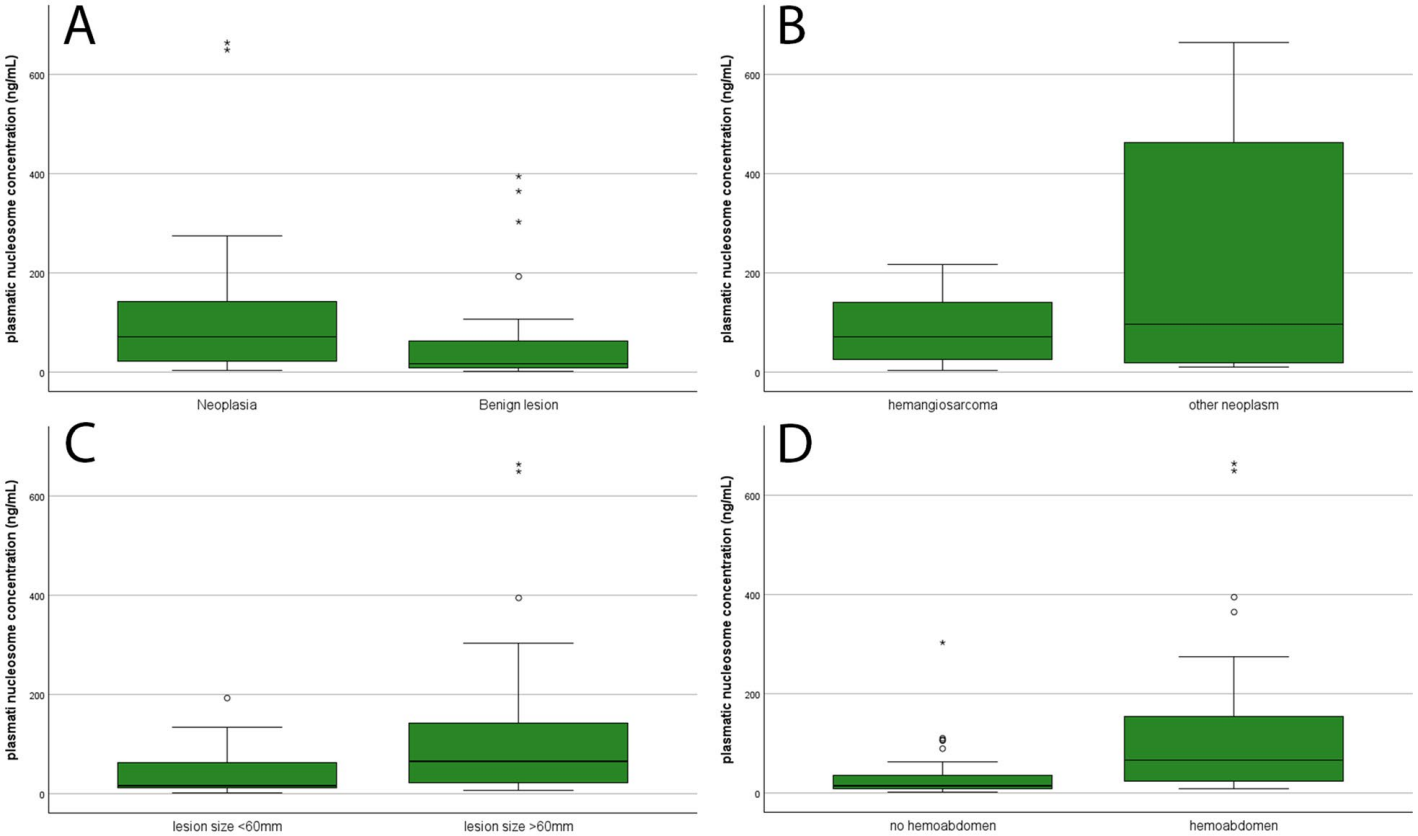
Box-plot representing plasmatic nucleosome concentration in 66 dogs with splenic nodular lesion based on the final diagnosis (A, B), the size of the lesion (C), or the presence of hemoabdomen (D). The boxes indicate the I-III interquartile range (IQR); horizontal lines indicate the median; vertical lines extend until the last value classified as “non-outlier”. ° = near-outlier (value further than 1.5*IQR from the III quartile). * = far-outlier (value further than 3.0*IQR from the III quartile).

The results of nucleosome concentration in different animal groups are detailed in Table 1 and Figure 1. The overall median nucleosome concentration was 26.9 ng/mL (range, 2.6-664.5 ng/mL). Dogs with splenic malignancy had a significantly higher nucleosome concentration compared to dogs with benign splenic nodular lesions (*p* = 0.001, Figure 1A). Since hemangiosarcoma was the most frequent diagnosis, a further comparison was made between nucleosome concentrations in dogs with splenic hemangiosarcoma and those with benign splenic nodular lesions, resulting in higher concentrations in dogs with hemangiosarcoma (*p* = 0.004). However, no significant differences were observed between dogs with hemangiosarcoma and those with other malignancies (*p* = 0.672, Figure 1B).

**Table 1.** Plasmatic nucleosome concentration in 66 dogs with splenic nodular lesion, according to the histopathological diagnosis.

Histopathological diagnosis	Number of dogs	Plasmatic nucleosome concentration (ng/mL)		
		Median	Minimum	Maximum
Benign lesion	38	16.5	2.6	395.2
Hemangiosarcoma	20	71.9	4.3	218.1
Other neoplasm	8	97.1	10.7	664.5

Out of the total, 55 (83.3%) dogs had a single splenic nodular lesion and 11 (16.7%) had multiple lesions. No significant differences were observed in the prevalence of single or multiple splenic nodular lesions between dogs with benign lesions and those with malignancy (*p* > 0.05). However, the latter group demonstrated significantly larger lesion size (*p* = 0.033). There were no differences in nucleosome concentration between dogs with single or multiple lesions (*p* = 0.690). Among dogs with a single splenic nodular lesion, the median lesion size was 60 mm (range, 6-250 mm). Nucleosome concentration exhibited a direct correlation with lesion size (*p* = 0.026) and was notably higher in dogs with lesions ≥60 mm compared to those with smaller lesions (*p* = 0.008) (Figure 1C).

Hemoabdomen was significantly more prevalent in dogs with splenic malignancy than in dogs with benign lesions (78.6% and 31.6%, respectively; *p* < 0.001). It was also more prevalent in dogs with hemangiosarcoma compared to those with benign splenic nodular lesions (85.0% vs. 31.6%, respectively; *p* < 0.001). No significant differences were observed in the occurrence of hemoabdomen between dogs with hemangiosarcoma and other malignancies (*p* > 0.05). The concentration of nucleosomes was higher in dogs with hemoabdomen (*p* < 0.001) compared to those without hemoabdomen (Figure 1D).

None of the variables investigated significantly influenced nucleosome concentration when dogs were stratified according to the presence or absence of hemoabdomen. Conversely, when dogs were stratified according to the lesion size, significantly higher nucleosome concentrations were found in dogs presenting with hemoabdomen (*p* = 0.022 for dogs with lesion <60 mm, and *p* = 0.027 for dogs with lesion ≥60 mm). Finally, among dogs with lesion size ≥60 mm, nucleosome concentration was significantly higher in cases of malignancy (*p* = 0.040).

**Figure 2.**
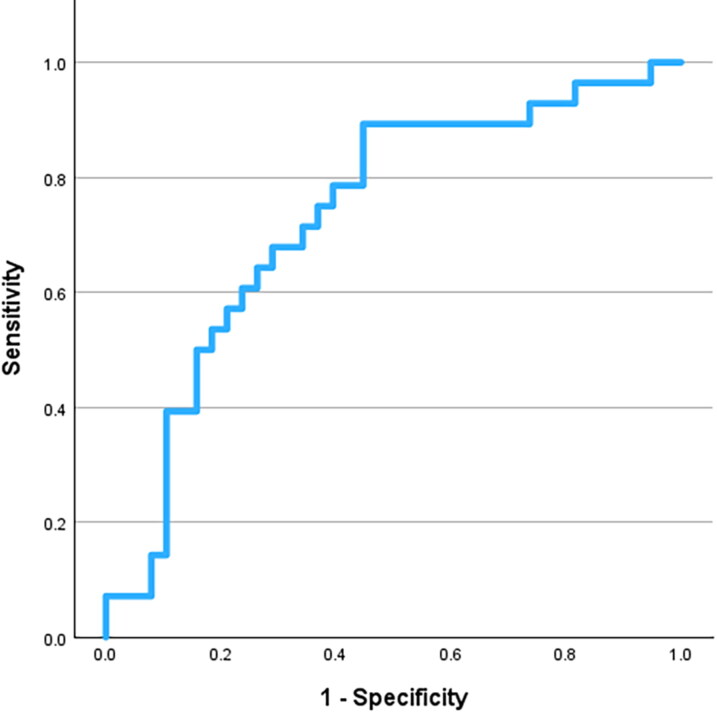
Receiving Operator Curve (ROC) drawn to assess the diagnostic performances of plasmatic nucleosome concentration to discriminate between benign and malignant splenic nodular lesions in 66 dogs.

Based on ROC curve coordinates, a cutoff of 50.0 ng/mL delivered the best overall sensitivity (67.9%) and specificity (71.1%) in identifying dogs with malignant lesions (Figure 2). Specifically, among 19 dogs with hemoabdomen, lesion size ≥60 mm and nucleosome concentration ≥50.0 ng/mL, 13 (68.4%) had splenic malignancies, whereas among 15 dogs without hemoabdomen, with lesion size <60 mm and with nucleosome concentration <50 ng/mL, 13 (86.7%) had benign splenic nodular lesions.

## Discussion

Previous studies have highlighted increased plasma nucleosome concentration in dogs with hemangiosarcoma (Wilson-Robles et al. 2021, 2022). However, those studies did not compare nucleosomes levels in dogs with splenic hemangiosarcoma and dogs with benign splenic nodular lesions nor with other malignancies that affect the spleen. This analysis is crucial to provide information on the diagnostic usefulness of nucleosomes level assessment. Moreover, nucleosome concentration has not been extensively studied in dogs with splenic nodular lesions with or without hemoabdomen.

Recognizing that nucleosome concentrations can be influenced by various factors, including processing time, sample type, and storage conditions (Wilson-Robles et al. 2020), we conducted a prospective study applying a meticulously standardized approach. Samples were collected, centrifuged and plasma was frozen before being shipped for analysis, maintaining a cold chain at −20 °C. This strategy aimed to minimize variability due to storage conditions, despite deviating from the manufacturer’s recommendations. Indeed, storage at −80 °C is recommended under the manufacturer’s instructions and according to published studies (Wilson-Robles et al. 2020). However, dogs with splenic nodular lesions, particularly those with hemoabdomen, are more likely to be treated in emergency care units where −80 °C freezers are seldom available, in contrast to academic units. Therefore, storage at −20 °C was decided upon because those freezers were accessible in all the different Institutions involved in the study. Storage temperature likely influenced our raw data, potentially explaining why we observed nucleosome concentrations lower than those previously reported for dogs with hemangiosarcoma (Wilson-Robles et al. 2021, 2022). This hypothesis was confirmed by our pilot study that compared nucleosome concentration between samples stored at −20 °C and −80 °C. Despite a good correlation, there was a significant proportional error, with nucleosome concentrations in samples stored at −20 °C being significantly lower compared to those stored at −80 °C. However, since the storage temperature was constant for all the samples in this study, differences among groups were likely not affected by the deviation from the manufacturer’s recommendations, or may even be underestimated, based on the presence of a negative proportional error by which the highest nucleosome concentrations were mostly affected by storage temperature. Based on these considerations, the results obtained here cannot be applied to samples stored at different temperature conditions.

Consistent with prior findings, dogs with hemangiosarcoma exhibited the highest elevation in nucleosome concentrations (Wilson-Robles et al. 2021, 2022). Diagnoses with increased nucleosome concentrations also included malignancies of other cellular origins. Noteworthy and as expected, median nucleosome concentrations in dogs with splenic malignancies were significantly higher when compared to benign splenic nodular lesions. This aligns with previous results in cancer-bearing dogs compared to healthy animals, where nucleosomes are released into the plasma at a higher rate during rapid cellular turnover and high cellular death rates (Holdenrieder et al. 2008; Dolan et al. 2021; Wilson-Robles et al. 2022). Similarly, the relatively lower concentrations of nucleosomes in dogs with benign splenic nodular lesions suggest that localized organ diseases of non-malignant origin may have minimal impact on circulating nucleosomes.

Stratifying malignant lesions based on histologic diagnosis revealed that dogs with hemangiosarcoma had significantly higher concentrations of nucleosomes compared to benign lesions, suggesting a potential diagnostic role of elevated nucleosomes for hemangiosarcoma within the spectrum of splenic lesions. Conversely, there were no significant differences in the plasma nucleosomes of dogs with hemangiosarcoma compared to those affected by other malignancies. Once again, this highlights the necessity for a multifaceted diagnostic approach that includes histopathology and IHC for the final classification of malignant splenic nodular lesions following splenectomy.

Elevated nucleosome concentration was correlated to both the presence of hemoabdomen and of hemangiosarcoma, underscoring the potential of nucleosome assessment in identifying those dogs with a higher likelihood of bearing malignant splenic nodular lesions or splenic rupture. Beside the high frequency of dogs with hemangiosarcoma presenting with ruptured spleen and hemoabdomen (Aronsohn et al. 2009), this scenario might also occur in dogs with splenic hematoma. In addition, it should be considered that hemorrhagic shock might lead to an increase in circulating plasma nucleosome concentrations, irrespective of the underlying malignancy. Of notice, the size of splenic nodular lesions also had an impact on nucleosome concentration. However, both the presence of hemoabdomen and the histopathologic diagnosis remained significant when dogs were stratified based on lesion size.

Based on our results, the ideal candidate for nucleosome concentration testing is more likely to be a healthy dog with an incidentally detected splenic nodular lesion smaller than 60 mm, rather than a dog presenting to emergency care with hemoabdomen. The cut-off we selected had disappointing sensitivity and specificity when applied to the whole study population. However, it correctly identified over 85% of dogs with benign lesions when applied to subjects without hemoabdomen and with smaller lesion sizes. The partial overlap of raw data between dogs with different diagnoses may account for this situation and should be taken into account during clinical decision-making. Moreover, it is worth noting that the turnaround time for the nucleosome detection approach described in this study, even under optimal conditions, is approximately 6 h. This limitation hinders its utility as a point-of-care test for emergent decision-making. However, since the initiation of the present study, an in-house point-of-care kit for the same test has been introduced in the market, thus significantly reducing the turnaround time.

Dogs without hemoabdomen do not need emergency care, allowing for ancillary tests before surgery and for adequate therapeutic planning. Various analyses can be included in the diagnostic workup for these patients, with cytology often representing a first-line test for a rapid and minimally invasive diagnosis (Yankin et al. 2020). However, splenic cytology faces some challenges, including variable diagnostic accuracy, with agreement with histopathology ranging from 37% to 100% (O’Keefe and Couto 1987; Braun and Hauser 2007; Christensen et al. 2009; Holter et al. 2023). Furthermore, cytology is not without risks, as it can potentially cause the rupture of any splenic lesion, leading to disease upstaging. In similar contexts, the evaluation of plasma nucleosome concentration emerges as a valuable tool in ruling out the possible malignant nature of the lesion. In our caseload, more than 85% of dogs without hemoabdomen, with <60 mm lesion size and displaying low nucleosome concentrations were diagnosed with benign splenic nodular lesions. While splenic histopathology remains necessary for the final diagnosis, clinicians should carefully weigh the pros and cons of this procedure, as histopathologic assessment usually follows splenectomy. This consideration is particularly crucial for dogs with a low probability of splenic cancer and concurrent diseases that may increase surgery complications and anesthetic risks. Nevertheless, the risk for splenic rupture also exists for benign lesions. Predicting that a lesion is likely benign and that surgery can be curative may increase owner compliance in authorizing splenectomy.

It is crucial to acknowledge that variations in nucleosome concentrations may also be associated with the dissemination of the disease or other comorbidities, such as diabetes mellitus, chronic kidney disease, pancreatitis, trauma, and infections (Penttilä et al. 2016; Phan et al. 2018; Goggs 2019; Lo Re et al. 2019). Since oncologic staging and diagnostic tests were not standardized among dogs included in the present study, we cannot completely exclude these factors as potential contributors to nucleosome concentration variations. However, dogs with severe systemic concomitant inflammatory diseases were not included in the study, to avoid possible influence on nucleosome concentrations (Letendre and Goggs 2018), whereas dogs with known chronic diseases were already receiving therapy, which is expected to reduce tissue damage and subsequent release of nucleosomes into circulation.

Several limitations in this study warrant acknowledgment. The low number of cases may impact statistical analyses and the generalizability of findings, emphasizing the need for larger cohorts, especially including a higher number of malignant splenic nodular lesion other than hemangiosarcoma, allowing for more stratified subgroup analyses. Sample storage conditions might have influenced nucleosome concentration in the samples. This detail precludes comparison with results already published on samples stored at −80° (Wilson-Robles et al. 2021, 2022), but expands the applicability of the test in the daily routine of small animal practice by reducing the limitations posed by storage at −80°.

In conclusion, while this study provides a foundational exploration of the potential diagnostic role of plasma nucleosome levels in canine splenic nodular lesions, to differentiate between malignant and benign ones, caution is needed in extrapolating results to a broader clinical context due to the mentioned limitations. Nucleosome detection, as described in this study, may not serve as a standalone diagnostic tool. Integration with other clinical parameters, imaging findings, and histopathology remains essential for a comprehensive diagnostic evaluation. Future investigations should include comprehensive clinical staging, conducting long-term follow-up assessments, and increasing the sample size to ensure robustness and generalizability of findings and to allow a comprehensive understanding the role of nucleosome as a prognostic biomarker. Despite these limitations, our study underscores the importance of ongoing research in this area to advance our understanding of novel diagnostic markers for canine splenic nodular lesions and improve clinical decision-making.

## Supplementary Material

Supplemental Material

## Data Availability

All data are available from the corresponding author, upon reasonable request.

## References

[CIT0001] Aronsohn MG, Dubiel B, Roberts B, Powers BE. 2009. Prognosis for acute nontraumatic hemoperitoneum in the dog: a retrospective analysis of 60 cases (2003–2006). J Am Anim Hosp Assoc. 45(2):72–77. doi:10.5326/0450072. 19258418

[CIT0002] Bao Y, Zhang D, Guo H, Ma W. 2024. Beyond blood: advancing the frontiers of liquid biopsy in oncology and personalized medicine. Cancer Sci. 115(4):1060–1072. doi:10.1111/cas.16097. 38308498 PMC11007055

[CIT0003] Braun AO, Hauser B. 2007. Correlation between cytopathology and histopathology of the skin, lymph node and spleen in 500 dogs and cats. Schweiz Arch Tierheilkd. 149(6):249–257. 17645034 10.1024/0036-7281.149.6.249

[CIT0004] Burti S, Zotti A, Bonsembiante F, Contiero B, Banzato T. 2022. A machine learning-based approach for classification of focal splenic lesions based on their CT features. Front Vet Sci. 9:872618. doi:10.3389/fvets.2022.872618. 35585859 PMC9108536

[CIT0006] Christensen N, Canfield P, Martin P, Krockenberger M, Spielman D, Bosward K. 2009. Cytopathological and histopathological diagnosis of canine splenic disorders. Aust Vet J. 87(5):175–181. doi:10.1111/j.1751-0813.2009.00421.x. 19382924

[CIT0007] Cleveland MJ, Casale S. 2016. Incidence of malignancy and outcomes for dogs undergoing splenectomy for incidentally detected nonruptured splenic nodules or masses: 105 cases (2009-2013). J Am Vet Med Assoc. 248(11):1267–1273. doi:10.2460/javma.248.11.1267. 27172343

[CIT0008] Cudney SE, Wayne AS, Rozanski EA. 2021. Diagnostic utility of abdominal ultrasonography for evaluation of dogs with nontraumatic hemoabdomen: 94 cases (2014–2017). J Am Vet Med Assoc. 258(3):290–294. doi:10.2460/javma.258.3.290. 33496618

[CIT0009] Dolan C, Miller T, Jill J, Terrell J, Kelly TK, Bygott T, Wilson-Robles H. 2021. Characterizing circulating nucleosomes in the plasma of dogs with lymphoma. BMC Vet Res. 17(1):276. doi:10.1186/s12917-021-02991-x. 34399763 PMC8365961

[CIT0010] Eberle N, von Babo V, Nolte I, Baumgärtner W, Betz D. 2012. Splenic masses in dogs. Part 1: epidemiologic, clinical characteristics as well as histopathologic diagnosis in 249 cases (2000–2011). Tierarztl Prax Ausg K Kleintiere Heimtiere. 40(4):250–260. 22911256

[CIT0011] Faroni E, Sabattini S, Guerra D, Iannuzzi C, Chalfon C, Agnoli C, Stefanello D, Polton G, Ramos S, Aralla M, et al. 2023. Timely adjuvant chemotherapy improves outcome in dogs with non-metastatic splenic hemangiosarcoma undergoing splenectomy. Vet Comp Oncol. 21(1):123–130. doi:10.1111/vco.12875. 36633399

[CIT0012] Fernandez S, Lang JM, Maritato KC. 2019. Evaluation of nodular splenic lesions in 370 small-breed dogs (<15 kg). J Am Anim Hosp Assoc. 55(4):201–209. doi:10.5326/JAAHA-MS-6934. 31099604

[CIT0013] Ferrari R, Marconato L, Boracchi P, Stefanello D, Godizzi F, Murgia D, Schievenin V, Amati M, Faroni E, Roccabianca P, et al. 2024. Splenic stromal sarcomas in dogs: outcome and clinicopathological prognostic factors in 32 cases. Vet Comp Oncol. 22(1):12–21. doi:10.1111/vco.12941. 37918913

[CIT0014] Flory A, Wilson-Robles H. 2024. Noninvasive blood-based cancer detection in veterinary medicine. Vet Clin North Am Small Anim Pract. 54(3):541–558. doi:10.1016/j.cvsm.2023.12.008. 38195361

[CIT0015] Goggs R. 2019. Effect of sample type on plasma concentrations of cell-free DNA and nucleosomes in dogs. Vet Rec Open. 6(1):e000357. doi:10.1136/vetreco-2019-000357. 31673376 PMC6802997

[CIT0016] Hammond TN, Pesillo-Crosby SA. 2008. Prevalence of hemangiosarcoma in anemic dogs with a splenic mass and hemoperitoneum requiring a transfusion: 71 cases (2003–2005). J Am Vet Med Assoc. 232(4):553–558. doi:10.2460/javma.232.4.553. 18279091

[CIT0017] Herman EJ, Stern AW, Fox RJ, Dark MJ. 2019. Understanding the efficiency of splenic hemangiosarcoma diagnosis using Monte Carlo simulations. Vet Pathol. 56(6):856–859. doi:10.1177/0300985819868732. 31422751

[CIT0018] Holdenrieder S, Nagel D, Schalhorn A, Heinemann V, Wilkowski R, von Pawel J, Raith H, Feldmann K, Kremer AE, Müller S, et al. 2008. Clinical relevance of circulating nucleosomes in cancer. Ann N Y Acad Sci. 1137(1):180–189. doi:10.1196/annals.1448.012. 18837945

[CIT0019] Holter DL, Nafe LA, Dugat DR, Hallman M, Ritchey JW, Fielder S, Rudra P. 2023. Diagnostic utility of ultrasound-guided fine-needle aspiration and needle-core biopsy sampling of canine splenic masses. Can J Vet Res. 87(4):265–271. 37790262 PMC10542949

[CIT0020] Kutara K, Seki M, Ishigaki K, Teshima K, Ishikawa C, Kagawa Y, Edamura K, Nakayama T, Asano K. 2017. Triple-phase helical computed tomography in dogs with solid splenic masses. J Vet Med Sci. 79(11):1870–1877. doi:10.1292/jvms.17-0253. 28993600 PMC5709567

[CIT0021] Letendre JA, Goggs R. 2018. Determining prognosis in canine sepsis by bedside measurement of cell-free DNA and nucleosomes. J Vet Emerg Crit Care (San Antonio). 28(6):503–511. doi:10.1111/vec.12773. 30299568

[CIT0022] Lo Re O, Maugeri A, Hruskova J, Jakubik J, Kucera J, Bienertova-Vasku J, Oben JA, Kubala L, Dvorakova A, Ciz M, et al. 2019. Obesity-induced nucleosome release predicts poor cardio-metabolic health. Clin Epigenetics. 12(1):2. doi:10.1186/s13148-019-0797-8. 31892362 PMC6938639

[CIT0023] Millar SL, Zersen KM. 2021. Diagnostic value of the ultrasonographic description of a splenic mass or nodule as cavitated in 106 dogs with nontraumatic hemoabdomen. Am J Vet Res. 82(12):970–974. doi:10.2460/ajvr.21.08.0130. 34714765

[CIT0024] Moore AS, Frimberger AE, Sullivan N, Moore PF. 2012. Histologic and immunohistochemical review of splenic fibrohistiocytic nodules in dogs. J Vet Intern Med. 26(5):1164–1168. doi:10.1111/j.1939-1676.2012.00986.x. 22882592

[CIT0025] O’Keefe DA, Couto CG. 1987. Fine-needle aspiration of the spleen as an aid in the diagnosis of splenomegaly. J Vet Intern Med. 1(3):102–109. doi:10.1111/j.1939-1676.1987.tb01997.x. 3506095

[CIT0026] Penttilä AK, Rouhiainen A, Kylänpää L, Mustonen H, Puolakkainen P, Rauvala H, Repo H. 2016. Circulating nucleosomes as predictive markers of sever acute pancreatitis. J Intensive Care. 4(1):14. doi:10.1186/s40560-016-0135-6. 26893906 PMC4758106

[CIT0027] Phan T, Mcmillan R, Skiadopoulos L, Walborn A, Hoppensteadt D, Fareed J, Bansal V. 2018. Elevated extracellular nucleosomes and their relevance to inflammation in stage 5 chronic kidney disease. Int Angiol. 37(5):419–426. doi:10.23736/S0392-9590.18.03987-1. 29644836 PMC6712971

[CIT0028] Sabattini S, Lopparelli RM, Rigillo A, Giantin M, Renzi A, Matteo C, Capitani O, Dacasto M, Mengoli M, Bettini G, et al. 2018. Canine splenic nodular lymphoid lesions: immunophenotyping, proliferative activity, and clonality assessment. Vet Pathol. 55(5):645–653. doi:10.1177/0300985818777035. 29807508

[CIT0029] Sabattini S, Rigillo A, Foiani G, Marconato L, Vascellari M, Greco A, Agnoli C, Annoni M, Melchiotti E, Campigli M, et al. 2022. Clinicopathologic features and biologic behavior of canine splenic nodules with stromal, histiocytic and lymphoid components. Front Vet Sci. 9:962685. doi:10.3389/fvets.2022.962685. 36032303 PMC9411940

[CIT0030] Schick AR, Grimes JA. 2022. Evaluation of the validity of the double two-thirds rule for diagnosing hemangiosarcoma in dogs with nontraumatic hemoperitoneum due to a ruptured splenic mass: a systematic review. J Am Vet Med Assoc. 261(1):69–73. 36322487 10.2460/javma.22.08.0389

[CIT0031] Wendelburg KM, Price LL, Burgess KE, Lyons JA, Lew FH, Berg J. 2015. Survival time of dogs with splenic hemangiosarcoma treated by splenectomy with or without adjuvant chemotherapy: 208 cases (2001–2012). J Am Vet Med Assoc. 247(4):393–403. doi:10.2460/javma.247.4.393. 26225611

[CIT0032] Wilson-Robles HM, Bygott T, Kelly TK, Miller TM, Miller P, Matsushita M, Terrell J, Bougoussa M, Butera T. 2022. Evaluation of plasma nucleosome concentrations in dogs with a variety of common cancers and in healthy dogs. BMC Vet Res. 18(1):329. doi:10.1186/s12917-022-03429-8. 36045415 PMC9429572

[CIT0033] Wilson-Robles H, Miller T, Jarvis J, Terrell J, Dewsbury N, Kelly T, Herzog M, Bygott T, Hardat N, Michel G, et al. 2020. Evaluation of nucleosome concentrations in healthy dogs and dogs with cancer. PLoS One. 15(8):e0236228. doi:10.1371/journal.pone.0236228. 32866177 PMC7458307

[CIT0034] Wilson-Robles H, Miller T, Jarvis J, Terrell J, Kelly TK, Bygott T, Bougoussa M. 2021. Characterizing circulating nucleosomes in the plasma of dogs with hemangiosarcoma. BMC Vet Res. 17(1):231. doi:10.1186/s12917-021-02934-6. 34187493 PMC8243913

[CIT0035] Wilson-Robles H, Warry E, Miller T, Jarvis J, Matsushita M, Miller P, Herzog M, Turatsinze J-V, Kelly TK, Butera ST, et al. 2023. Monitoring plasma nucleosome concentrations to measure disease response and progression in dogs with hematopoietic malignancies. PLoS One. 18(5):e0281796. doi:10.1371/journal.pone.0281796. 37163491 PMC10171669

[CIT0036] Wood CA, Moore AS, Gliatto JM, Ablin LA, Berg RJ, Rand WM. 1998. Prognosis for dogs with stage I or II splenic hemangiosarcoma treated by splenectomy alone: 32 cases (1991–1993). J Am Anim Hosp Assoc. 34(5):417–421. doi:10.5326/15473317-34-5-417. 9728473

[CIT0037] Yankin I, Nemanic S, Funes S, de Morais H, Gorman E, Ruaux C. 2020. Clinical relevance of splenic nodules or heterogeneous splenic parenchyma assessed by cytologic evaluation of fine-needle samples in 125 dogs (2011–2015). J Vet Intern Med. 34(1):125–131. doi:10.1111/jvim.15648. 31692075 PMC6979272

