# Supplementary material for: Circulating nucleosomes as a potential cancer biomarker in dogs with splenic nodular lesions
Source: Vet Q. 2024 Sep 3;44(1):1–7. doi: 10.1080/01652176.2024.2399648 (PMC11373370; doi:10.1080/01652176.2024.2399648)
Supplement: Supplemental Material [file TVEQ_A_2399648_SM2537.pdf]

SUPPLEMENTARY TABLE 1: clinical data of 66 dogs with splenic nodular lesion

Case nr	breed	sex	age (years)	weight (kgs)	number of lesions	lesion size (mm)	hemoabdomen	concomitant disease	diagnosis
1	pure	neutered male	9	38.3	single	6	no	none	not neoplastic
2	pure	neutered male	15	5.2	single	10	no	none	not neoplastic
3	pure	male	11	19.3	single	12	no	none	not neoplastic
4	mixed	male	10	19.6	single	13	no	none	not neoplastic
5	mixed	spayed female	11	21.7	single	16	no	none	not neoplastic
6	pure	neutered male	11	57	single	20	no	chronic kidney disease	not neoplastic
7	mixed	spayed female	13	14	single	20	no	none	not neoplastic
8	mixed	spayed female	8	25	single	25	no	none	not neoplastic
9	mixed	female	12	14.7	single	25	no	hypothyroidism	not neoplastic
10	pure	spayed female	8	7.5	single	30	no	none	not neoplastic
11	mixed	male	16	15	single	30	no	none	not neoplastic
12	pure	neutered male	12	22.1	single	30	no	congestive heart failure	not neoplastic
13	mixed	neutered male	12	36	single	30	yes	none	not neoplastic
14	pure	female	12	12	single	40	no	dermatopathy	not neoplastic
15	mixed	spayed female	12	19.6	single	45	no	none	not neoplastic
16	pure	male	10	5.8	single	45	yes	none	not neoplastic
17	mixed	spayed female	8	16.7	single	50	no	none	not neoplastic
18	pure	spayed female	11	32	single	50	no	none	not neoplastic
19	pure	male	8	50	single	50	yes	none	not neoplastic
20	pure	neutered male	11	8	single	50	yes	none	not neoplastic
21	pure	male	8	32.3	single	60	no	none	not neoplastic
22	pure	neutered male	12	18	single	60	no	none	not neoplastic
23	pure	neutered male	15	8.5	single	60	yes	none	not neoplastic
24	mixed	neutered male	11	50	single	80	no	none	not neoplastic
25	pure	male	11	35	single	80	yes	none	not neoplastic
26	mixed	spayed female	11	10.5	single	80	yes	none	not neoplastic
27	pure	male	6	34.5	single	90	no	none	not neoplastic
28	pure	male	11	38	single	114	yes	none	not neoplastic
29	mixed	neutered male	12	22.3	single	120	yes	none	not neoplastic
30	pure	female	7	7	single	120	no	none	not neoplastic
31	pure	male	10	7	single	120	yes	none	not neoplastic
32	pure	male	8	31.2	single	128	no	none	not neoplastic
33	pure	male	7	43	single	160	yes	none	not neoplastic
34	pure	male	9	31.1	single	180	no	none	not neoplastic
35	mixed	male	12	11	multiple		no	none	not neoplastic
36	mixed	spayed female	9	20	multiple		yes	none	not neoplastic

37	mixed	spayed female	10	15	multiple		no	none	not neoplastic
38	pure	spayed female	12	20.5	multiple		no	hypothyroidism	not neoplastic
39	pure	spayed female	12	21.3	single	20	yes	gastritis	hemangiosarcoma
40	mixed	male	10	43.6	single	30	yes	none	hemangiosarcoma
41	pure	neutered male	11	33	single	40	no	chronic lymphocytic leukemia	hemangiosarcoma
42	pure	spayed female	7	35	single	50	yes	none	hemangiosarcoma
43	pure	female	10	36.3	single	60	yes	none	hemangiosarcoma
44	pure	male	11	36	single	60	yes	none	hemangiosarcoma
45	mixed	spayed female	9	27	single	70	yes	none	hemangiosarcoma
46	mixed	neutered male	14	35	single	70	yes	none	hemangiosarcoma
47	pure	female	11	16	single	80	yes	Addison disease	hemangiosarcoma
48	mixed	female	9	26.2	single	87	yes	none	hemangiosarcoma
49	mixed	male	13	45	single	97	no	none	hemangiosarcoma
50	mixed	spayed female	15	18	single	100	yes	none	hemangiosarcoma
51	pure	spayed female	10	39	single	100	yes	none	hemangiosarcoma
52	pure	spayed female	9	40	single	150	yes	none	hemangiosarcoma
53	pure	spayed female	9	34	single	180	no	none	hemangiosarcoma
54	mixed	male	12	10	multiple		yes	none	hemangiosarcoma
55	pure	spayed female	11	40	multiple		yes	none	hemangiosarcoma
56	pure	male	8	35	multiple		yes	none	hemangiosarcoma
57	mixed	male	10	32.1	multiple		yes	none	hemangiosarcoma
58	pure	female	12	12	multiple		yes	none	hemangiosarcoma
59	pure	female	14	32.2	single	25	yes	none	other neoplasia
60	pure	female	14	32.8	single	52	no	urinary tract infection	other neoplasia
61	mixed	male	15	12.1	single	80	yes	none	other neoplasia
62	pure	neutered male	11	35	single	100	yes	none	other neoplasia
63	mixed	spayed female	7	22.8	single	150	yes	congestive heart failure	other neoplasia
64	mixed	spayed female	10	27.7	single	250	no	dermatopathy and epilectic seizures	other neoplasia
65	mixed	female	13	15	multiple		no	none	other neoplasia
66	pure	neutered male	12	39	multiple		yes	none	other neoplasia
